# Myocarditis in Humans and in Experimental Animal Models

**DOI:** 10.3389/fcvm.2019.00064

**Published:** 2019-05-16

**Authors:** Przemysław Błyszczuk

**Affiliations:** ^1^Department of Clinical Immunology, Jagiellonian University Medical College, Cracow, Poland; ^2^Department of Rheumatology, Center of Experimental Rheumatology, University Hospital Zurich, Zurich, Switzerland

**Keywords:** myocarditis, animal models, coxsackievirus B3, Chagas disease, heart-specific autoimmunity, experimental autoimmune myocarditis

## Abstract

Myocarditis is defined as an inflammation of the cardiac muscle. In humans, various infectious and non-infectious triggers induce myocarditis with a broad spectrum of histological presentations and clinical symptoms of the disease. Myocarditis often resolves spontaneously, but some patients develop heart failure and require organ transplantation. The need to understand cellular and molecular mechanisms of inflammatory heart diseases led to the development of mouse models for experimental myocarditis. It has been shown that pathogenic agents inducing myocarditis in humans can often trigger the disease in mice. Due to multiple etiologies of inflammatory heart diseases in humans, a number of different experimental approaches have been developed to induce myocarditis in mice. Accordingly, experimental myocarditis in mice can be induced by infection with cardiotropic agents, such as coxsackievirus B3 and protozoan parasite *Trypanosoma cruzi* or by activating autoimmune responses against heart-specific antigens. In certain models, myocarditis is followed by the phenotype of dilated cardiomyopathy and the end stage of heart failure. This review describes the most commonly used mouse models of experimental myocarditis with a focus on the role of the innate and adaptive immune systems in induction and progression of the disease. The review discusses also advantages and limitations of individual mouse models in the context of the clinical manifestation and the course of the disease in humans. Finally, animal-free alternatives in myocarditis research are outlined.

## Introduction

The World Heart Federation estimated that about 400,000 persons die annually worldwide because of inflammatory heart diseases. Epidemiologic post-mortem studies identified myocarditis as an important cause of unexpected and sudden death. Myocarditis has been implicated in cardiac sudden deaths in young adults at the rate of 8.6–12% (1, 2). The occurrence of myocarditis in sudden death in children was reported at the rate of 17% (3). Considering cardiovascular death in children and young adults, myocarditis was a major cause in 10–42% cases (4).

Myocarditis is classified as an inflammatory disease of the heart muscle. Traditionally, myocarditis is diagnosed based on endomyocardial biopsies. According to the widely used “Dallas” criteria published in 1987, a diagnosis of active myocarditis requires the presence of inflammatory infiltrates of non-ischemic origin in myocardial tissue associated with necrosis and/or degeneration of adjacent cardiomyocytes. Presence of inflammatory infiltrates in the absence of apparent myocyte damage is classified as borderline myocarditis (5). The definition of myocarditis has been more recently enumerated by the ESC Working Group on Myocardial and Pericardial Diseases, which proposed abnormal number of inflammatory infiltrates in myocardial tissue as ≥14 leucocytes/mm^2^ including up to 4 monocytes/mm^2^ with the presence of ≥7 cells/mm^2^ CD3-positive T lymphocytes (6). These criteria have become widely accepted. Beside this common histological feature of inflammatory condition of the heart, there is a high diversity observed in the disease cause, characteristic of inflammatory infiltrates, clinical symptoms, course of inflammation, and the prognosis.

## Pathophysiology of Myocarditis in Human

### From Myocarditis to Dilated Cardiomyopathy

Patients with myocarditis, proven via biopsies, show 55–80% 5-year transplantation-free survival (7–10). Mortality is observed not only during the acute phase, but also during the follow up. Myocarditis is a progressive disease with two post-acute clinical scenarios. In the first scenario, resolution of the inflammation is followed by complete recovery associated with improved heart function. It has been reported that myocarditis resolves spontaneously in ~40–60% of cases (11, 12). In the second scenario, the acute phase is followed by development of stable dilated cardiomyopathy (DCM). Follow-up clinical studies showed development of DCM pathology over a period of several years in 14–52% patients with histologically proven myocarditis (12). DCM is referred to as left ventricular dilation associated with systolic dysfunction in the absence of coronary artery disease. Histologically, DCM is manifested by the extensive replacement of cardiac muscle cells with fibrotic tissue and deposition of collagen (13, 14). DCM patients develop not only heart pump weakening, but also heart valve problems, blood clots, and arrhythmias leading to heart and secondary organ failures. DCM patients show transplantation-free survival at the rate of 50–60% over 5 years in children (15) and in adults (7, 16, 17), however improved medication can increase the survival rate up to 80% (16). Particularly high mortality has been reported for patients with DCM due to Chagas disease (17). DCM can be associated with inflammation of the myocardium. Co-occurrence of myocarditis and DCM is referred to as inflammatory DCM (iDCM) (6). In fact, 16–30% of patients with chronic DCM show immunohistochemical evidences of myocardial inflammation (15, 18). Cardiac deaths in the follow-up myocarditis cohort were predominantly associated with DCM characteristic including systolic dysfunction and left ventricular dilation (9). Accordingly, ventricular dilatation and systolic dysfunction are associated with worse prognosis for myocarditis patients (8).

#### Disease Etiology

In Europe and North America myocarditis is often idiopathic. Infections with cardiotropic enteroviruses such as coxsackievirus B3 (CVB3) have been associated with the disease and considered as a causative agent. The prevalence of enteroviruses detected in cardiac biopsies of myocarditis patients was reported at the rate of 14–57% (19). Other viruses such as parvovirus B19, adenoviruses or herpesviruses have also been detected in biopsies of myocarditis patients (19). Over decades, a shift in detection of enterovirus and adenovirus to parvovirus B19 and herpesviruses has been observed. However, the causative role of detected viruses in myocarditis patients is not evident. For example, surprisingly high prevalence of parvovirus B19 has been detected in myocarditis patients, suggesting its pathogenic role in the disease (20). More recent data showed, however, a high prevalence of parvovirus B19 also in myocarditis-negative hearts (21). Thus, the causative or associative role of individual viral infections in pathogenesis of myocarditis is still under investigation. Furthermore, it also remains to be elucidated, whether the persistence of the viral genome in the myocardium influences the clinical outcomes. So far, clinical studies resulted rather in contradicting conclusions (8, 22). In Europe and North America, myocarditis is also diagnosed in patients with Lyme disease (borreliosis). The disease is caused by bacteria *Borrelia burgdorferi*, which is transmitted by the bite of an infected ticks. It is estimated that up to 10% of Lyme disease patients develop myocarditis (23).

In Latin America, infections with protozoan *Trypanosoma cruzi* (Chagas disease) are the most common cause of inflammatory heart disease (24). The etiology of Chagas disease is quite well-established. Bites of blood-sucking triatomines (called also kissing bugs) spread the infective forms of the parasite. In humans, trypanosomal infection triggers the disease with two clinically distinct phases. The acute phase lasts several weeks and is usually asymptomatic or is associated with fever and local swelling or skin lesion. 10–30 years later about one third of the infected individuals develop a chronic form of the disease primarily manifested by DCM or iDCM, but also by neurological and/or gastrointestinal track pathologies. The chronic phase of the Chagas disease is usually progressive, leading to permanent heart failure (25). Cardiac dysfunction due to myocarditis and iDCM represents the most frequent and the most severe clinical manifestation of Chagas disease, which is associated with poor prognosis and high mortality rates (24, 26).

Non-infectious causes of myocarditis include mainly systemic autoimmune diseases and certain medications (27). Myocarditis has been observed for example in systemic lupus erythematosus (28) and in myasthenia gravis (29) patients. Recently, numerous cases of fatal myocarditis have been reported in cancer patients shortly after starting treatment with immune checkpoint inhibitors (30, 31). Immune checkpoint inhibitors refer to a category of drugs (antibodies) targeting negative regulators of T cell response, such as cytotoxic T-lymphocyte associated protein-4 (CTLA-4), programmed cell death protein-1 (PD-1), and PD-1 ligand (PD-L1). It is considered that immune checkpoint inhibitors may activate heart-specific autoimmunity in predisposed individuals (32). Today, it is widely accepted that autoimmune mechanisms are involved in the development and/or progression of myocarditis (33). Clinical evidences suggest active autoimmune response in human myocarditis on both, cellular and humoral levels. Initially, the concept of heart-specific autoimmunity came from the observation of high titers of heart-specific autoantibodies in CVB3 infected individuals. Accordingly, 30% of patients with myocarditis and DCM develop high titers of heart-specific autoantibodies (34). Cardiac myosin heavy chain (MyHC) has been identified as the most prominent autoantigen for circulating heart autoantibodies in myocarditis and cardiomyopathy patients (35). In fact, the presence of anti-MyHC autoantibodies has been associated with worse left ventricular systolic function and diastolic stiffness in patients with chronic myocarditis (36). There are strong indications that also antigen-presenting cells play an important role in the pathogenesis of myocarditis in humans by promoting autoimmune mechanisms. For example, histological analysis demonstrated increased levels of major histocompatibility complex (MHC) class I and II, known as human leukocyte antigen (HLA) complexes (37) and co-stimulatory molecules B7-1, B7-2, and CD40 (38) in hearts of myocarditis patients. More recent data pointed also to the importance of the humoral response in myocarditis (39).

### Clinical Assessment and Classifications of Myocarditis

#### Diagnosis of Myocarditis

Clinical manifestation of myocarditis varies with a broad spectrum of symptoms, ranging from asymptomatic courses through shortness of breath, cardiac arrhythmias to chest pain resembling myocardial infarction (27, 40, 41). Myocarditis is often associated with left ventricular dysfunction (42), in some cases with cardiac arrhythmias (43) and elevated levels of certain biomarkers (6). These clinical symptoms are, however, not specific for myocarditis and the definitive diagnosis requires detection of inflammatory cells in the myocardium, typically on endomyocardial biopsy. In addition to histological analyses of cardiac biopsies, the assessment of myocarditis could be performed using a cardiac magnetic resonance imaging (44, 45). Improved imaging protocols confirmed usefulness of this modern, non-invasive technology in diagnosing myocarditis (46). Magnetic resonance imaging shows excellent diagnostic accuracy in patients with acute symptoms, while its usefulness is limited in patients with suspected chronic myocarditis (45, 47). On the one hand, histological evaluation of myocardial biopsies still represents a “gold standard,” mainly because it allows not only to diagnose myocarditis, but also to identify infective agents and characterize the type of inflammatory cells. These data can be indicative for selection of the personalized treatment strategy and may be predictive for disease outcome (27, 40, 41, 48). In fact, endomyocardial biopsies confirm inflammation in 44–70% of patients with suspected myocarditis (49–51). On the other hand, due to the often patchy pattern of inflammation in the heart, endomyocardial biopsies-based diagnosis of myocarditis yields rather low sensitivity (52–54). Biopsies guided by non-invasive molecular imaging and/or electroanatomic mapping could increase the success rate. It seems, however, that the actual prevalence of myocarditis possibly remains underestimated. It is noteworthy that, incidental inflammation of the myocardium evaluated in a clinicopathological study reported that any inflammatory cells were present in 18% and multifocal inflammation in 9% of total cardiac and non-cardiac deaths (55).

#### Clinical Classifications of Myocarditis

In the clinic, myocarditis can be classified based on the causative, histological, and clinicopathological criteria, which are summarized in Table 1. The causative criteria define infectious agents (virus, protozoa, or bacteria) or non-infectious condition (autoimmune diseases, medications etc.) associated with myocarditis. Identification of the infectious agent or potential non-infectious trigger may be indicative not only for disease etiology, but also helps to choose the most effective therapeutic strategy for the affected patients. In addition to identification of the causative agent, histological and immunohistological analyses are performed to categorize myocarditis based on the presence, morphology and type of inflammatory infiltrates in the myocardium. Lymphocytic myocarditis characterized by extensive infiltration of lymphocytes and monocytes with signs of cardiomyocyte necrosis (active lymphocytic myocarditis) represents the most frequent type of myocarditis (10). Lymphocytic myocarditis is often observed in myocardium tested positive for viral persistence. Less common forms of myocarditis represent giant cell myocarditis and eosinophilic myocarditis. Giant cell myocarditis is characterized by the presence of multinucleated giant cells and lymphocytes on heart biopsies. Presence of giant cells within non-caseating granulomas, usually associated with myocardial fibrosis is referred to as cardiac sarcoidosis (56). The characteristic feature of eosinophilic myocarditis is the presence of eosinophil-rich infiltrates in the myocardium and extensive myocyte necrosis, which is accompanied with elevated level of circulating eosinophils (57). Giant cell myocarditis and eosinophilic myocarditis are associated with particularly poor prognosis (57–60).

**Table 1:** Clinical classifications of myocarditis.

**Causative criteria**	**Histological criteria**	**Clinicopathological criteria**
**Virus:** coxsackievirus B3, adenoviruses or herpesviruses and other	**Active myocarditis:** cardiac inflammation with apparent cardiomyocyte necrosis	**Fulminant myocarditis:** sudden onset, severe heart failure, cardiogenic shock or life-threatening arrhythmias
**Protozoa:** *Trypanosoma cruzi* (Chagas disease)	**Borderline myocarditis:** cardiac inflammation without evident cardiomyocyte necrosis	**Acute myocarditis:** highly variable from asymptomatic to cardiogenic shock, ventricular dysfunction, may progress to dilated cardiomyopathy
**Bacteria:** *Borrelia burgdorferi* (Lyme disease) and other	**Lymphocytic myocarditis:** extensive infiltration of lymphocytes and monocytes	**Chronic active myocarditis:** variable clinical symptoms, ventricular dysfunction, relapses of clinical symptoms and chronic myocardial inflammation on histology
**Immune checkpoint inhibitors:** anti-CTLA-4, anti-PD-1 or anti-PD-L1 therapy	**Giant cell myocarditis:** multinucleated giant cells and lymphocytes on heart biopsies	**Chronic persistent myocarditis:** persistent histologic infiltrate with myocyte necrosis, chest pain or palpitation without ventricular dysfunction
**Systemic autoimmune diseases:** Systemic lupus erythematosus, myasthenia gravis and other	**Eosinophilic myocarditis:** eosinophil-rich infiltrates with extensive myocyte necrosis	

Combination of the histologic data and clinical course of the disease resulted in clinicopathologic classification of myocarditis (61). Parameters such as onset of the disease, initial clinical and histological presentation, disease course and cardiac dysfunction define acute, fulminant, chronic active and chronic persistent subtypes of myocarditis. Acute myocarditis represents the most common type of myocarditis, in which symptoms last typically for days or weeks and the acute phase is followed by spontaneous improvement or development of stable DCM (62). In patients with fulminant myocarditis disease progresses rapidly resulting in severe heart failure and cardiogenic shock with mortality rate of 30–40% during the acute phase (63, 64). Patients diagnosed with fulminant myocarditis surviving the acute phase have been instead suggested to have excellent long-term prognosis (65), although a recently published study demonstrated contradictive findings (66). In its chronic form, myocarditis is detected over a period of three or more months. Clinical and histologic relapses and development of ventricular dysfunction is characteristic for chronic active myocarditis, whereas chronic persistent myocarditis is characterized by persistent presence of inflammatory cells in the myocardium, but it is usually not associated with ventricular dysfunction.

## Mouse Models of Experimental Myocarditis

The need to understand cellular and molecular mechanisms of inflammatory heart diseases led to development of animal models for experimental myocarditis. In general, these models can be categorized based on the causative agents into two major classes: infectious and non-infectious. In infectious models, pathogens associated with myocarditis in humans are used to induce cardiac inflammation in animals. CVB3 and *T. cruzi* represent two classical infectious pathogens used for induction of experimental myocarditis in mice. In non-infectious models, myocarditis is typically triggered by an autoimmune response against heart-specific antigens. A comparative summary of the selected models is presented in the Table 2.

**Table 2:** Characteristics of commonly used mouse models of experimental myocarditis.

**Mouse model**	**Susceptible mouse strains**	**Histological characteristic**	**Clinicopathological characteristic**	**Advantages and limitations**	**References**
*In vitro*-passaged CVB3 or EMCV (10^3^-10^5^ TCID_50_ or PFU)	BALB/c, A/J, DBA-2, C57BL/6 (4-9 weeks old)	Active myocarditis	Acute myocarditis	(+) use of clinically relevant virus (+) suitable to study CVB3 replication (–) high mortality (–) high biosafety standards required	(67–82)
Heart-passaged CVB3 (10^3^-5x10^5^ PFU)	BALB/c, A/J, C57BL/6 (4-9 weeks old)	Active lymphocytic myocarditis, fibrosis	Acute myocarditis (C57BL/6) and chronic active myocarditis (BALB/c, A/J)	(+) use of clinically relevant virus (+) allows to study disease progression (–) involvement of immune system in CVB3 clearance and autoimmunity (–) high biosafety standards required	(83–94)
Reovirus or MAV-1 (10^4^-10^7^ PFU)	BALB/c, C57BL/6, Swiss (2-7 days old)	Active myocarditis	Acute myocarditis	(+) suitable to study viral replication (+) unique model of pediatric myocarditis (–) clinically irrelevant viruses (–) non-standard methodologies required	(95–98)
*T. cruzi* infection (50 – 10^6^ trypomastigotes)	BALB/c, A/J, C57BL/6, DBA-2, C3H/He, Swiss (4-12 weeks old)	Active lymphocytic myocarditis, fibrosis	Chronic active myocarditis	(+) use of clinically relevant pathogen (+) recapitulate course of Chagas disease (–) pathogen strain-dependent variability (–) long-term model	(99–133)
Immunization with α-MyHC or troponin I peptide and CFA	BALB/c, A/J, A.SW (6-8 weeks old)	Active/borderline lymphocytic or eosinophilic^*^ myocarditis, fibrosis	Acute myocarditis progressing to DCM	(+) biosafe (+) suitable to study transition from myocarditis to DCM (–) non-physiological disease induction (–) immunization with CFA	(134–163)
Delivery of bmDCs loaded with α-MyHC peptide	BALB/c (6-8 weeks old)	Borderline lymphocytic myocarditis	Acute myocarditis	(+) biosafe (+) suitable to study dendritic cells (–) non-physiological disease induction (–) culture of bmDCs in FCS-rich medium	(145, 164, 165)
TCR-M transgenic mice	BALB/c (≥4 weeks old)	Active/borderline lymphocytic myocarditis	Chronic persistent myocarditis	(+) biosafe (+) suitable to study pathophysiology of heart-specific T cells (–) non-physiological disease induction (–) lack of heart non-specific T cells	(166)
CMy-mOVA mice injected with OT-I CD8^+^ effector T cells (2.5 × 10^4^-3 × 10^6^)	C57BL/6 (6-20 weeks old)	Active lymphocytic myocarditis	Fulminant myocarditis	(+) biosafe (+) suitable to study T cell-mediated cytotoxicity against cardiomyocytes (–) reactivity against non-cardiac antigen (–) *in vitro* T cell activation	(167–170)
PD-1/PD-L1-deficiency	BALB/c, MRL (≥4 weeks old)	Active/borderline lymphocytic myocarditis	Fulminant myocarditis	(+) biosafe (+) suitable to study side effects of anti-PD-1/PD-1L therapy (–) multiorgan involvement (–) high mortality	(171–175)
HLA-DQ8 transgenic mice	BALB/c, NOD (≥4 weeks old)	Active/borderline lymphocytic myocarditis	Fulminant myocarditis	(+) biosafe (+) suitable to study cardiac antigen presentation (–) human-mouse chimeric system (–) high mortality	(176–180)

*α-MyHC, myosin heavy chain α; bmDCs, bone marrow-derived dendritic cells; CFA, complete Freund's adjuvant; CMy-mOVA - cardiac myocyte restricted membrane-bound ovalbumin; CVB3, coxsackievirus B3; EMCV, encephalomyocarditis virus; FCS, fetal calf serum; HLA, human leukocyte antigen; MAV-1, murine adenovirus type 1; OT-I, major histocompatibility complex class I-restricted ovalbumin-specific; PD-1, programmed cell death protein-1; PD-L1, PD-1 ligand; PFU, plaque forming unit; TCID_50_, 50% tissue culture infectious dose; TCR-M, T cell receptor (TCR) specific to α-MyHC*.

* *in Ifng^−/−^Il17a^−/−^ BALB/c mice*.

### Viral Models of Experimental Myocarditis

#### Experimental Myocarditis Induced With CVB3

CVB3 has been implicated to the pathogenesis of myocarditis in humans and therefore this virus was used to induce experimental myocarditis in animals. Coxackieviruses belonging to the *Picornaviridae* family represent positive-sense single-stranded RNA enteroviruses. Coxsackieviruses are typically transmitted by the oral route and for replication require host cells. Unlike other serotypes, CVB3 efficiently infects and replicates in cardiomyocytes leading to their death through apoptosis (181) or necroptosis (182). Effective CVB3 replication has been demonstrated also in cardiac fibroblasts (183). CVB3 infection begins by coupling the virus with host-cell coxsackievirus and adenovirus receptor (CAR), and decay-accelerating factor (DAF). Additionally, recent data pointed to the relevance of NOD2 in CVB3 uptake (184). After entering into the cytoplasm, viral RNA is translated and then transcribed. The viral genome is further translated into viral structural proteins, which assemble with the positive-strand RNA viral genome forming the complete infectious virion (185). Infected cardiomyocytes become ultimately lysed, which results in release of cytosolic proteins and virus progeny. Active viral replication as well as latent viral persistence have been described in hearts of myocarditis patients (186).

The first successful myocarditis induction in mice using purified CVB3 (Nancy strain) was reported in 1974 (67). The Nancy strain of CVB3 is the most commonly used virus to induce myocarditis in mice until today. The virus was passaged *in vitro* in the host cells. Inoculation of purified, *in vitro*-passaged CVB3 resulted in high viral replication in hearts of host mice. This model is characterized by substantial cardiomyocyte necrosis, moderate inflammation, pancreatitis, and often high mortality during the acute phase of disease in BALB/c, A/J, and C57BL/6 mice (67–74). Poor survival rate of mice infected with *in vitro*-passaged CVB3 led to development of the heart-passaged CVB3 model of experimental myocarditis (83). In this model, hearts of mice infected with CVB3 were used for preparation of the infective pathogen. Such heart-passaged CVB3 containing not only the virus, but also cardiac myosin is inoculated into host animals. In this model, viral replication peaks around day 7 and the pathogen is cleared around day 14, post-infection. Infected mice develop acute myocarditis around day 10–14, which is characterized by massive infiltration of cardiac tissue with primarily cells of myeloid lineage accompanied by T (mainly CD4^+^) and some B lymphocytes in various mouse strains including BALB/c, A/J, ABY/SnJ, and C57BL/6 (83–89). In this model myocarditis is associated with left ventricular dysfunction during the acute phase. In contrast to high mortality rate observed in *in vitro*-passaged CVB3 model, typically all mice infected with heart-passaged CVB3 survive.

Following the acute myocarditis phase, disease course strongly depends on the genetic background of infected mice. Susceptible BALB/c, ABY/SnJ, and A/J mice progress to a phenotype of iDCM, characterized by chronic myocarditis, myocardial fibrosis, and cardiomyopathy, which is observed at day 28 post-infection and later (83, 89, 90). Whereas, mice with C57BL/6 genetic background do not develop DCM/iDCM phenotype (83, 91, 92), unless they are additionally treated with lipopolysaccharide (LPS) (93, 94). Infection with CVB3 leads to impaired cardiac functionality at later stages, which develops independently of the fibrotic phenotype in the heart (92, 94).

Infection with CVB3 triggers the respective innate and adaptive immune responses. Synthesis of antiviral cytokines such as type I interferons (IFNs) represent the first line of the innate immune defense against CVB3 infection, which aims to inhibit viral replication. Accordingly, treatments with IFN-α or IFN-β were reported to effectively eliminate virus in CVB3 infected mice (75) as well as in myocarditis patients (187). Following CVB3 entry into the target cell, the virus can engage intracellular nucleotide binding and oligomerization domain (NOD)-like receptors (NLRs) and activate certain Toll-like receptors (TLRs) (188). Activation of TRIF-dependent TLR3 has been recognized to be crucial for antiviral type I IFN production (76, 77). Interestingly, activation of other NLR and TLR pathways exacerbate myocarditis in CVB3 infected mice through negative regulation of type I IFN and stimulation of proinflammatory cytokines (73, 74, 184).

The innate immune response is usually followed by the adaptive response against the infective virus. In CVB3-mediated experimental myocarditis, the protective role of the adaptive immune response has been well-established. Studies using immunodeficient mice showed that lack of T and B cells led to viral persistence and enhanced myocarditis upon CVB3 infection (189, 190). Interestingly, in the CVB3 myocarditis model CD4^+^, but not CD8^+^ T cells play a pivotal role in viral clearance and thus protect infected mice from persistent cardiac inflammation (191, 192), whilst CD8^+^ T cells have been implicated mainly in the autoimmune response (191, 193). These paradoxical observations can be explained by the findings that CD4^+^ T cell response recognizes infected, but not uninfected myocytes, while CD8^+^ effector T cells react only to uninfected myocytes through recognition of cardiac myosin (193). Furthermore, natural killer (NK) (78) and NK T cells (79, 80) were also reported to play protective roles in CVB3-induced myocarditis.

As stated above, heart-specific autoimmunity has been implicated in the pathogenesis of viral myocarditis. Early data indeed pointed to the development of functional heart-specific autoimmune response in CVB3 infected mice (81). Detection of circulating autoantibodies represents a basic diagnostic assay indicating ongoing autoimmune disorder. Following CVB3 infection, high titers of heart-specific autoantibodies have been detected in host A/J and BALB/c mice (194, 195). Heart-specific autoantibodies are commonly detected also in myocarditis patients (196) pointing to similarity between mouse models and clinical scenario. Infection with CVB3 activates also cardiac myosin reactive CD4^+^ T cells in mice (69). Data from experimental model provided evidences that cardiac myosin reactive cells functionally contribute to cardiac pathology during chronic stage of the disease in CVB3 infected BALB/c mice (197). Heart specific autoimmunity seems to be a consequence of significant release of cardiac peptides from cardiomyocytes lysed during cardiotropic infection or molecular mimicry (epitope cross-reactivity) between the virus and cardiac proteins. Cardiac autoantigens in the presence of certain co-stimulatory, so called “third signal” cytokines can trigger the effector response of autoreactive T lymphocytes. It has been suggested that proinflammatory cytokines, mainly TNFα and IL-1β, produced during the innate response against viral infection play critical role in induction of the effector autoimmune response (33). Thus, myocarditis is likely a result of not only immune response against the infective virus, but also a consequence of boosted heart-specific autoimmune response. It seems that viral infection primary triggers myocarditis, while autoimmune response contributes to disease progression. In summary, myocarditis and iDCM phenotypes following CVB3 infection is the result of interplay between immune responses against the virus and heart-specific autoimmunity. Published data indicate that CVB3 infectious myocarditis mouse models accurately recapitulate principles of the immune responses in humans.

#### Experimental Myocarditis Induced With Other Viruses

CVB3 represents the most common, but not the only virus used for induction of experimental myocarditis in mice. Cardiac inflammation associated with cardiomyocyte necrosis can be also induced with encephalomyocarditis virus (EMCV) (82). EMCV, like CVB3, are positive single-stranded RNA viruses belonging to the *Picornaviridae* family, which induce necrotic myocarditis with the similar mechanism of action (198). Enteroviruses CVB3 and EMVC are used to induce myocarditis in ≥4 weeks old mice. Whereas, the murine adenovirus type 1 (MAV-1) and reovirus 8B have been used to establish the mouse model for pediatric myocarditis. Depending on the delivery route, MAV-1 induces lethal [intraperitoneal injection (95)] or non-lethal [intranasal infection (96)] myocarditis in newborn mice. Infection of newborn mice with reovirus 8B also induces acute viral myocarditis. In this model myocarditis is characterized by marked cardiomyocyte necrosis and mild inflammation leading to death of infected BALB/c (97), but not C57BL/6 (98) mice. In contrast to the CVB3 model, autoimmunity seems not to be involved in myocarditis progression in reovirus 8B infected mice (97). Myocarditis in children is a deadly disease, particularly for newborns and infants and viral infections have been suggested as important causative agents in these young patients (199, 200). In that respect, MAV-1 and reovirus B8 models could be useful to study pathophysiological mechanisms of the disease in children.

### Experimental Models of Chagas Heart Disease

Trypanosomal infection can cause myocarditis and iDCM in mouse organisms. Experimental Chagas heart disease has been successfully established in a number of mouse lines using various *T. cruzi* strains including Colombian, Tulahuen, CL Brener Brazil, the Y, and SylvioX10, but so far, no model has been generally accepted as the classical one. Pathogenic trypanosome strains were isolated from Chagastic patients, insect vectors, and animal reservoir (99). *Trypanosoma cruzi* is typically passaged in mice and bloodstream trypomastigotes (infective form of the parasite) are transferred into experimental animals by different delivery routes including intraperitoneal, intradermal, and oral transmission. Inbred strains BALB/c, C57BL/6, A/J, DBA-2, or C3H/He are often used as hosts, however many laboratories use outbred Swiss mice to induce experimental Chagas heart disease. Disease course, organ involvement and survival rate in different models are characterized by high variability and strongly depend on the *T. cruzi* strain, delivery route and genetic background of the recipient mice (99–105). An example of the high variability in mouse Chagastic model was demonstrated in the experiment with Swiss mice infected with different clones of the Colombian strain, which showed mouse mortality ranging from 0 to 100% depending on the clone (102). Apparently, interplay between the host, parasite genetics and environmental factors ultimately determine the outcome of a mouse infection with *T. cruzi*. Trypanosomal infection may lead to myocarditis development in recipient mice within 1–3 weeks post-infection (101, 102). In the chronic form, experimental Chagas heart disease is associated with progressive inflammation, iDCM phenotype and heart dysfunction. This phenotype is observed several months post-infection (100, 104, 106–110). It seems that chronic models recapitulate not only the end stage heart phenotype, but also the course of the disease observed in Chagastic patients.

During the acute phase of Chagas disease, trypomastigotes spread with the bloodstream throughout the body and enter into target cells, in which they differentiate into amastigotes and multiply causing death of the host cells. Infection of myeloid cells and cardiomyocytes represent two important check points for the progression of the disease. Myeloid cells, like macrophages and dendritic cells actively internalize parasites by phagocytosis. The innate immune response of macrophages and dendritic cells represent the first line of defense against the parasite involving TLR-dependent and TLR-independent mechanisms. Trypomastigote cell surface membrane glycosylphosphatidylinositol-anchored mucin-like glycoproteins and glycoinositolphospholipids as well as secreted Tc52 proteins activate innate immune cells through TLR2-, TLR4-, and TLR9-dependent mechanisms (111–113). The classical TLR-dependent response activates NF-κb and MAPK pathways leading to production of proinflammatory cytokines including TNFα and Th1 polarizing IL-12. Such responses are indeed observed during trypanosomal infections in mice (114, 115). Furthermore, in response to IFN-γ (produced by Th1 cells, but also by activated NK cells) and TNFα macrophages produce nitric oxide. This short-lived free radical effectively suppresses parasite replication and represents the primary defense mechanism during the acute phase of the infection (116). During the acute trypanosomal infection, Th1 polarization is facilitated also through TLR-independent innate mechanisms. For example, a cysteine protease cruzipain released by trypomastigote generates short-lived kinins, which stimulate IL-12 production through the bradykinin B_2_ receptor on the host cells and subsequently induce the protective Th1 response in infected mice (117).

Furthermore, *T. cruzi* triggers a robust adaptive immune response in the infected mouse organism. Phenotypically, infected mice show accumulation of lymphocytes in the spleen and subcutaneous lymph nodes associated with thymus atrophy (118). Early reports demonstrated persistent, non-specific polyclonal activation of T and B cells with phenotypic hypergammaglobulinemia (119, 120). Indeed, *T. cruzi* components such as DNA or glycoproteins have been shown to non-specifically activate T and B cells (121, 122), whereas more recent data pointed to the key role of antigen-specific response during parasite infection in mouse and in human (123, 124, 201). Importantly, the adaptive immune response plays a crucial role in pathogen clearance. Depletion of CD4^+^ or CD8^+^ T cells leads to an increase in parasite burden and exacerbation of myocarditis (125). Similarly, B cells and trypanosoma-specific antibodies have been shown to protect infected mice from uncontrolled parasite replication (126–128). Furthermore, interplay between T and B cells is needed for the effective adaptive immune response against trypanosomal infection (127, 128).

During the acute phase of the disease, the immune response eventually eliminates most of the infective pathogens, but not all. It has been suggested that a certain degree of parasite persistence, particularly in cardiac tissue correlates with the development of DCM phenotype and heart failure. Trypanosomal reactivation is commonly observed under immunosuppressive conditions in mouse models (129) and in humans (202) supporting the concept of parasite persistence. In the clinical scenario, Chagastic patients are treated with one of two anti-parasitic medications, benznidazole or nifurtimox, which generate free radicals, killing *T. cruzi* pathogens. In the chronic model of experimental Chagas heart disease, elimination of the pathogen during post-acute phase with benznidazole was shown to prevent development of severe chronic DCM in infected mice (110, 130). These results indicate that chronic experimental model can recapitulate incomplete eradication of *T. cruzi* observed in Chagastic patients. It is important to note that effectiveness of the anti-parasitic treatment decreases as the disease progresses. Ultimately, treatment with benznidazole fails to improve cardiac clinical outcomes in Chagastic patients with established DCM (203).

Heart-specific autoimmunity has been suggested as another disease progressing factor in Chagas heart disease. Heart-specific T cells and high titers of heart-specific autoantibodies have been identified in experimental mouse models (131, 204) as well as in Chagastic patients (205). In *T. cruzi* infected mice, development of heart-specific autoimmunity is associated with the genetic background of the host organism. Prominent humoral and cellular anti-cardiac myosin responses develop in A/J and BALB/c, but not in C57BL/6 mice (132, 206). Such an anti-cardiac myosin autoimmune response was shown to be non-essential for development of the acute phase of myocarditis (133), but has been implicated in the progression of post-acute cardiomyopathy during chronic phase of experimental Chagas heart disease (132). In mouse model of Chagas disease, the adaptive immune response, which plays a crucial role in the host defense against the infecting parasite, seems to contribute also to disease progression.

### Mouse Models of Experimental Autoimmune Myocarditis

As presented above, clinical observations and experimental data from infectious models provide strong evidences for involvement of autoimmune mechanisms in the development and progression of myocarditis. In infectious models, T and B cells are primary involved in pathogen clearance. It is therefore practically impossible to uncouple the defense from autoimmune mechanisms using commonly available technologies. The need to understand contribution and molecular mechanisms of autoimmunity led to development of rodent models of experimental autoimmune myocarditis (EAM), in which myocarditis is induced by heart-reactive T cells in the absence of infectious pathogen.

In the context of autoimmune myocarditis, the question arises whether or not heart-specific T cells naturally occur in mouse and in human. In principle, vertebrates are protected from autoreactive T cells by the immune tolerance mechanisms. In the thymus, central tolerance specifically eliminates newly developing T cells, recognizing body's own antigens in a process called “negative selection.” In this process presentation of self-antigens by antigen-presenting medullary cells is essential for maintenance of a central tolerance. Surprisingly, α-isoform of MyHC (α-MyHC), unlike other cardiac proteins, is not expressed in cells implicated in T cell tolerance. This results in undisturbed development of α-MyHC-specific T cells and leads to their physiological presence in the periphery in mice and in human (207). Thus, α-MyHC represents the major cardiac self-antigen. In fact, many currently used EAM models take advantage of this and activate naturally existing α-MyHC-specific T cells in order to trigger autoimmune-mediated myocarditis. Data from experimental animal models clearly demonstrated that autoreactive CD4^+^ T lymphocytes were able to trigger myocarditis and DCM.

#### The “Classical” Model of Experimental Autoimmune Myocarditis

The first attempt to induce heart-specific autoimmunity in animals was reported in 1958 (208), but in 1987 Neu et al. published the basis for the currently most commonly used mouse model of EAM (134). In this publication, authors demonstrated that delivery of cardiac myosin together with the complete Freund's adjuvant (CFA) induced myocarditis with high prevalence and high titers of myosin autoantibodies in genetically predisposed mice (134). Currently, in this “classical” model of EAM susceptible mice are immunized with α-MyHC peptide together with CFA at day 0 and 7. Myocarditis in α-MyHC/CFA immunized mice is characterized by massive infiltration of mainly myeloid cells together with CD4^+^ T cells and few B and CD8^+^ T lymphocytes. Inflammation of cardiac tissue occurs typically 14–21 days after the first immunization. Resolution of the inflammation is followed by the progressive accumulation of fibrotic tissue in the myocardium, ventricular dilatation and impaired heart function in some mice (135–146). Thus, this model allows to study not only autoimmune mechanisms, but also transition from myocarditis to DCM phenotype. Of note, α-MyHC/CFA immunization of *Ifng*^−/−^*Il17a*^−/−^ mice results in myocarditis with extensive infiltration of eosinophils in the myocardium representing a unique model of eosinophilic myocarditis (147).

Published data point to a central role of CD4^+^ T cells in the α-MyHC/CFA model. Depletion of CD4^+^ T cells prevents induction of myocarditis and the adoptive transfer of purified CD4^+^ T cells from immunized mice successfully transfers the disease into immunodeficient hosts (148, 149). A simple passive transfer of high-titer myosin autoantibodies failed to transfer myocarditis in the recipient mice (150), however monoclonal anti-myosin antibodies were shown to induce myocarditis in a predisposed mouse strain (151). CD8^+^ T cells, instead, contribute mainly to myocarditis severity, but are not essential for disease induction (148). However, recent data showed that using the specific α-MyHC peptide for EAM induction, CD8^+^ T cells were able to limited extends convey myocarditis (152).

Co-delivery of a strong adjuvant, such as CFA represents the second key element of EAM induction in the “classical” model. CFA contains heat-killed *Mycobacterium tuberculosis*, which can activate TLR2, TLR4, and TLR9 on host cells (209). Activation of TLRs on the innate immune cells triggers secretion of a broad range of cytokines. In the adaptive immune response, the “third signal” cytokines produced by dendritic cells program vitality and expansion potential of antigen-activated T lymphocytes (210). The “third signal” cytokines have been also shown to polarize differentiating T cells toward Th1, Th2, or Th17 lineages. The importance of the “third signal” cytokines in the development of EAM has been demonstrated in a number of studies. Genetic deletion or blockage of the “third signal” cytokine signaling, including TNF-α (153, 154), GM-CSF (155), IL-1 (156), IL-6 (157), or IL-23 (158) resulted in complete resistance or amelioration of EAM. It remains, however, unclear whether the acute response to adjuvant in mice reflects immune processes during myocarditis induction in humans.

Development of EAM is a multifactorial process, which depends not only on the presence of heart-specific T cells and TLR activation, but also strongly on genetic predisposition. α-MyHC/CFA immunization induces myocarditis in susceptible strains only. Mice on BALB/c, A/J or A.SW background are susceptible to EAM, while mice on C57BL/6 background are resistant (134, 159–161). From a practical point of view, the resistance to EAM of widely-used C57BL/6 strain limits use of numerous transgenic models without the need for back-crossing onto the susceptible background. Differences in MHC haplotypes (H-2) of susceptible and resistant strains have been suggested to determine susceptibility of mice to EAM (148, 162). However, differences in susceptibility of A.SW and B10.S mice, which share the same H-2 genes, pointed also to the importance of non-H-2 genes in EAM development (163). Summarizing, the “classical” EAM model offers a well-established, simple and safe method to study heart-specific autoimmunity and progression of cardiac inflammation to DCM phenotype, but is limited to few inbred strains only.

#### Other Models of Experimental Autoimmune Myocarditis

The idea that activation of self-antigen presenting cells is critical for myocarditis induction led to development of “dendritic cell” EAM model. It has been demonstrated that myocarditis could be effectively induced by adoptive transfer of bone marrow-derived dendritic cells (bmDCs) loaded with α-MyHC peptide and activated with LPS - a major component of the outer membrane of Gram-negative bacteria and the anti-CD40 antibody. In the “dendritic cell” EAM model, adoptive transfer of activated α-MyHC-loaded bmDCs at days 0, 3 and 5 results in acute myocarditis at days 8–12 (145, 164, 165). In contrast to the “classical” model, mice receiving α-MyHC-loaded bmDCs develop moderate fibrosis on the follow up. However, additional administration of CFA significantly accelerates fibrosis and induces ventricular dilatation and heart dysfunction in this model (211).

The “classical” and the “dendritic cell” EAM models, both rely on activation of naturally existing α-MyHC-reactive CD4^+^ T cells. Non-transgenic mice contain physiologically very low prevalence of α-MyHC-reactive T cells and TLRs stimulation with CFA or LPS is needed not only for polarization, but also for expansion of activated α-MyHC-reactive T helper cells. High prevalence of these autoreactive cells can be alternatively obtained by transgenic overexpression of T cell receptor (TCR) specific to α-MyHC (TCR-M). A consequence of the high number of circulating α-MyHC-reactive T cells in the TCR-M transgenic mice is spontaneous development of progressive myocarditis associated with ventricular wall thickening, but without evident systolic dysfunction (166). The TCR-M transgenic model is particularly useful to study pathogenesis of autoreactive T cells in the absence of exogenous TLR agonists.

In contrast to widely studied CD4^+^ T cells, the role of CD8^+^ T cells (known also as cytotoxic T lymphocytes) in heart-specific autoimmunity is less understood. Unlike CD4^+^ T cells, CD8^+^ T cells recognize antigens presented by MHC class I molecules and directly induce apoptosis of antigen presenting cells by secreting cytotoxins, such as perforins and granzymes. In order to develop CD8^+^ T cell-dependent myocarditis model, transgenic mice (on C57BL/6 genetic background) expressing cardiomyocyte-restricted membrane-bound ovalbumin (OVA) were injected with *in vitro* expanded and polarized MHC class I-restricted, OVA-specific OT-I CD8^+^ T cells. Adoptive transfer of high doses (≥5 × 10^5^) of effector OT-I CD8^+^ T cells caused massive cardiomyocyte cell death associated with lymphocytic (both CD4^+^ and CD8^+^) and myeloid cell infiltration. Severe myocarditis caused death of affected mice 3–7 days post T cell transfer (167, 168). In this model, T cell polarizing factors, such as IL-12 (167) and T-bet (169) played a key role in disease pathogenesis. Disease severity in this model correlates with the number of injected lymphocytes. In contrast to high doses, low doses (2.5 × 10^4^) of effector OT-I CD8^+^ T cells induce a transient and moderate myocarditis only (170). This model is particularly useful to study CD8^+^ T cell-mediated cytotoxicity against cardiomyocytes. Reactivity against non-physiological antigen (OVA) seems to be, however, a major drawback of this method.

Under homeostatic conditions, effector functions of CD4^+^ and CD8^+^ T cells are controlled by regulatory T cells (Treg). Accordingly, adoptive transfer of Treg-depleted T cells induces multiorgan inflammation including fatal autoimmune myocarditis and high-titer anti-myosin autoantibodies in the recipient mice. Of note, inflamed myocardium displayed multinucleated inflammatory cells resembling giant cell myocarditis in humans (212).

Once T cells become activated, more regulatory mechanisms control their expansion and effector function. Immune checkpoint regulators, such as PD-1 represents an example of regulatory mechanisms. Mechanistically, PD-1-PD-1L signaling inhibits TCR signaling on activated T cells and thus suppresses autoimmune response. Accordingly, mice deficient of PD-1 or PD-1L spontaneously develop systemic or organ-specific inflammations. Progressive myocarditis, iDCM phenotype and heart failures have been described in PD-1- and PD-1L-deficient mice on BALB/c (171, 172) and MRL (173, 174) genetic backgrounds, although for BALB/c not in all housing conditions (175). In mice lacking PD-1, fatal myocarditis caused high mortality in particular on MRL genetic background. Cardiac inflammation in PD-1-deficient mice is entirely dependent on the adaptive immunity (autoimmunity) and could be transferred by splenocytes (171, 174). In mouse models, PD-1-PD-1L signaling protects from myocarditis mediated by CD4^+^ T (175) and by CD8^+^ T (170) cells. PD-1 deficiency results in myocarditis also in aged C57BL/6 mice, but these mice are characterized by multiorgan inflammation and represent rather a model of systemic lupus erythematosus (213). It seems that the use of PD-1- and PD-1L-deficient mice developing progressive myocarditis represents a suitable model to study mechanisms of cardiac side effects observed during anti-PD-1-PD-1L therapies in oncological patients.

Although α-MyHC represents a main cardioimmunogenic antigen, other cardiac proteins can also trigger heart-specific autoimmunity. Immunization of A/J mice with recombinant cardiac troponin I peptide together with CFA induces myocarditis, which is followed by myocardial fibrosis, ventricular dilatation, and impaired systolic function (214). Unlike α-MyHC, troponin I is expressed in medullary thymic epithelial cells (207). The occurrence of circulating troponin I-reactive T cells is, instead, a result of inefficient elimination of autoreactive T cells during the “negative selection.” The “leakage” of autoreactive T cells into periphery is a physiological phenomenon and its degree depends on the affinity of the TCR to the antigen-presenting medullary cells. Clinical data show elevated levels of troponin I in around one third of myocarditis patients (215), but high titers of anti-troponin I antibodies were detected only in 7% of DCM patients (216). It seems that troponin I represents rather a secondary autoantigen in heart-specific autoimmunity in myocarditis patients.

Presentation of cardiac antigen represent another important element of autoimmunity. Clinical studies suggested that specific HLA haplotypes are associated with heart-specific autoimmunity. This idea was functionally confirmed by introducing specific HLA complexes into a mouse. Replacement of mouse MHC class II with the specific HLA-DQ8 in NOD or BALB/c mice resulted in spontaneous development of myocarditis and iDCM phenotype (without evident fibrosis), and were associated with cardiac arrhythmias and high mortality of the transgenic mice (176–180). This model resembles a course of fatal fulminant myocarditis in humans. Adoptive transfer experiments pointed to the key role of CD4^+^ T cells in pathogenesis of the disease in this mouse model (178, 179). Interestingly, introduction of HLA-DR3 or HLA-DQ6 failed to induce cardiac pathology pointing the specific role of HLA-DQ8 in heart-specific autoimmunity (177, 178). The HLA-DQ8 transgenic mice seem to be particular useful to study mechanisms of cardiac antigen presentation and induction of heart-specific autoimmunity.

## A Triphasic Model of Myocarditis

**Figure 1:**
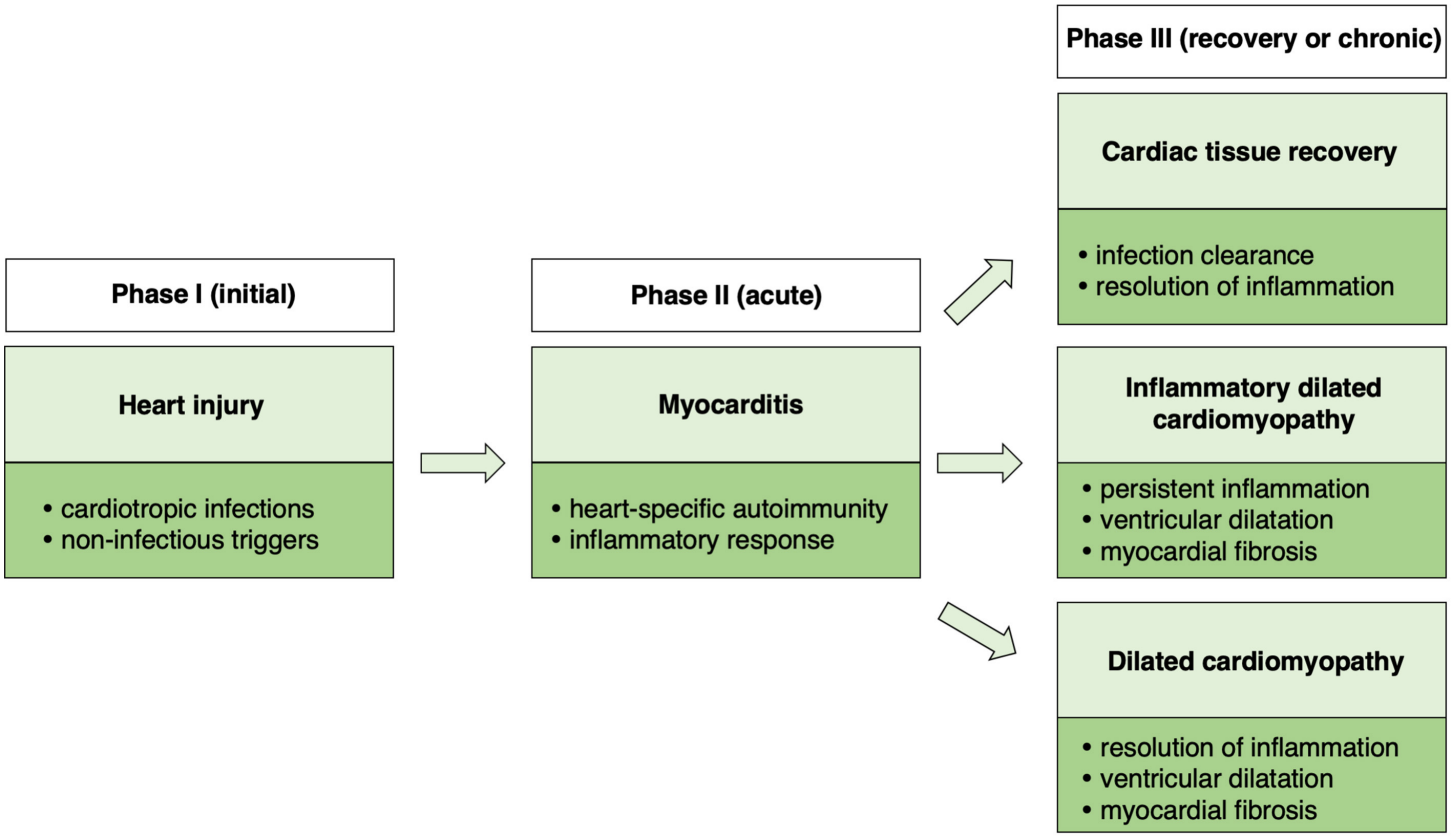
Schematic presentation of a triphasic model of myocarditis.

Taking together clinical observations as well as data from animal models, a triphasic model of myocarditis development and progression could be proposed (Figure 1). The initial phase is associated with heart injury, caused usually by cardiotropic infections or non-infectious triggers. Damaged myocardium induces primary inflammatory response and development of heart-specific autoimmunity, which results in the development of myocarditis. In certain cases, myocarditis can be directly induced by heart-specific autoimmunity. Cardiac inflammation can be transient or chronic. In the transient form, acute phase of myocarditis is followed by complete resolution of inflammation or development of stable DCM. In case of chronic myocarditis, many patients develop also DCM phenotype (iDCM). Cardiac dysfunction in DCM and in iDCM is in most cases progressive leading to end stage organ failure.

## Which Model of Experimental Myocarditis to Choose?

As presented above, a number of different animal models of myocarditis have been developed. Experimental myocarditis in mice can be induced with the whole spectrum of disease triggers ranging from clinically relevant agents, such as infective pathogens up to artificial models like transgenic animals. Depending on the model, myocarditis is characterized by different composition of inflammatory infiltrates and different extent of necrotic myocardium. In many models, DCM or iDCM phenotype associated with heart dysfunction represent the end stage of the disease. The question arises, which model reflects human myocarditis in the most relevant way. As myocarditis in humans shows high diversity in terms of causative agents, autoimmune response, course of inflammation, and progression to DCM/iDCM, it becomes evident that one animal model cannot mirror all aspects of the disease in humans.

For modeling of myocarditis with known etiology, as for example CVB3-mediated myocarditis or Chagas disease, use of the same infectious agent seems to be the most obvious choice. Infectious pathogens spread and induce myocarditis in mice and in humans using similar mechanisms, therefore these models are particularly useful to study early phase of disease. However, it is difficult to uncouple immune mechanisms involved in pathogen clearance and in autoimmune responses. Furthermore, handling the human infective pathogens is potentially dangerous for experimentators and requires exceptionally high biosafety standards.

In contrast, non-infectious models are safe for experimentators, but induce myocarditis in rather non-physiologic way. Nevertheless, lack of infectious agents allows to better study autoimmune mechanisms and involvement of the immune system to progression of myocarditis to DCM/iDCM phenotype. Summarizing, the usefulness of the respective mouse model is mostly limited to certain aspects of the disease in humans. It is therefore very important to address scientific question by choosing the relevant model.

## Alternatives for Animal Studies in Myocarditis Research

In developed countries, public approval to conduct animal experimentations is today low as never before. Activists raise ethical concerns and appeal to minimize or even to stop performing experiments involving animals. Some of these postulates pointing to protocol optimization and to reduction of severity in animal experimentation are rational and have been already implemented in the form of 3R (replacement, reduction, and refinement) guidelines, but is there a realistic animal-free alternative in myocarditis research?

It seems that certain aspects of the disease, for example replication of cardiotropic pathogens, fibroblast-to-myofibroblast transition, endothelial cell activation, can be effectively addressed using conventional *ex vivo* or *in vitro* systems. These systems are suitable for molecular studies, but rather poorly reflect biomechanical and biochemical microenvironment of cardiac tissue. Dynamic development of three-dimensional cell culture methodologies, like organoids or microtissues represents a recent advancement, which can address some of these concerns. Furthermore, development of the human induced pluripotent stem cell (iPSC) technology represents another important milestone toward animal-free research. The use of human iPSC-derived cardiomyocytes in combination with other cell types allows today for a simplistic modeling of a human heart (217, 218). Furthermore, *de novo* tissue fabrication opens new possibilities to integrate bioscaffolds for improved architecture and microelectronics for live monitoring of cardiac tissue (219). These advances offer potentially very attractive replacements for animal models (220). However, generation of physiologically-relevant human cardiac tissue faces a number of serious problems, which include immature state of iPSC-derived cardiomyocytes, limited availability of non-cardiac primary cells (fibroblasts, endothelial cells) and high costs of *de novo* tissue fabrication. Moreover, in myocarditis active migration of inflammatory cells into the tissue plays a key role in the disease development. Currently, modeling of inflammatory response in terms of influx of inflammatory cells into cardiac tissue *in vitro* is not available.

Summarizing, it seems that the whole process of myocarditis is too complex to reproduce it outside of a body by using today's technologies. However, certain aspects of the disease can be reliably studied *in vitro* and upcoming advances may allow to reduce animal research in the future.

## Conclusive remarks

Animal models represent an important platform in preclinical research. As presented above, developed models of experimental myocarditis appear to reliably mirror many specific aspects of the disease in humans. Currently, these animal models are commonly used to get an insight into pathophysiology of myocarditis on molecular and cellular level and to test pharmaceutic compounds for treatment efficacy and safety. However, animal studies are commonly unicenter, involving mostly low group sizes and experiments are rarely reproduced by others. Furthermore, in some cases, published papers lack detailed description of used methodologies. Thus, the power of such exploratory studies is usually low.

In laboratory practice, typical large scale, multicenter animal studies are not possible due to ethical and economic reasons. It seems that synthetic research integrating data from independent studies is needed to increase the power of experimental findings. In this case, the use of the same or similar procedures is a basic prerequisite to analyse data from different laboratories. In the area of experimental myocarditis, some models show high reproducibility in terms of used protocols and obtained results. In particularly, published data by independent laboratories on disease course and severity of myocarditis induced in specific inbred strains by infection with CVB3 or by immunization with α-MyHC/CFA show high consistency. In contrast, other models like experimental Chagas disease show high variability due to inconsistencies in methodologies. The use of standardized procedures would allow to more effectively plan experimentations and more accurately interpret obtained data. Furthermore, the use of unified methodologies would be important to effectively share omics resources and to implement meta-analyses in animal research. Combined efforts are therefore needed to more efficiently use the potential of animal models in order to translate this knowledge into innovative, more effective treatment therapies.

## Author Contributions

The author confirms being the sole contributor of this work and has approved it for publication.

### Conflict of Interest Statement

The author declares that the research was conducted in the absence of any commercial or financial relationships that could be construed as a potential conflict of interest.
